# Supplementary material for: Current Status and Barriers of Exercise in Individuals with Spinal Cord Injuries in Korea: A Survey
Source: Healthcare (Basel). 2024 May 16;12(10):1030. doi: 10.3390/healthcare12101030 (PMC11121255; doi:10.3390/healthcare12101030)
Supplement: Supplementary file 1 [file healthcare-12-01030-s001.zip › healthcare-2954441-supplementary.pdf]

Questions		Response values
Personal information	Gender	1: Male<br>2: Female
	Age (years)	1: 19–28<br>2: 29–38<br>3: 39–48<br>4: 49–58<br>5: 59–68<br>6: Over 69
	Marital status	1: Single<br>2: Married<br>3: Cohabiting<br>4: Divorced or separated<br>5: Widowed
	Household composition	1: Live alone<br>2: Live with more than two people
	Income level<br>(Million Korean Won)	1: Under 2<br>2: 2~3.99<br>3: 4~5.99<br>4: 6~7.99<br>5: 8~9.99<br>6: over 10
Injury characteristics and health status	Date of injury	1: Under 3 months<br>2: 3 months ~ 6 months<br>3: 6 months ~ 1 year<br>4: 1 year ~ 5 years<br>5: 5 years ~ 10 years<br>6: Over 10
	Type of injury	1: Trauma<br>2: Disease
	Level of injury	1: Cervical<br>2: Thoracic<br>3: Lumbar<br>4: Sacral
	ASIA Impairment Scale	1: A<br>2: B<br>3: C<br>4: D
	Impact of complication on daily life	1: No problem at all<br>2: Slightly problematic<br>3: Average<br>4: Severely problematic

		5: Very severely problematic	
	Impact of underlying disease on daily life	1: No problem at all<br>2: Slightly problematic<br>3: Average<br>4: Severely problematic<br>5: Very severely problematic	
	Pain score	1–10	
	Smoking	1: Yes<br>2: No	
	Drinking	1: Yes<br>2: No	
	BMI (kg/m^2)		
Physical condition and quality of life	Subjective health perception	1: Very good<br>2: Good<br>3: Fair<br>4: Poor<br>5: Very poor	
	Mobility independence		
	Urination		
	Defecation		
	Self-care		
Activity and participation	Activities of daily living	1–5	
	Mobility	1–5	
	Function and participation	1–5	
Status of exercise and requirements	Have you performed self-exercise within the past 3 months?	1: Yes	When did you start exercising after injury?
			What is the intensity of exercise?
			What is the frequency of your exercise?
			How many minutes do you exercise per workout?
			Where do you exercise?
		2: No	What barriers are stopping you from exercising?
	Have you used a community exercise facility?	1: Yes	How was your satisfaction?
		2: No	Why haven't you tried it? What are the barriers to you?
	What support do you want to provide to address barriers?	1: Accurate diagnosis of patient condition and determination of exercise intensity<br>2: Development of exercise plans and provision of programs by stage<br>3: Provision of exercise related welfare information and linkage<br>4: Transportation support for visits to exercise facilities<br>5: Rental of exercise equipment	

	6: Coordination with professional trainers<br>7: etc.
